# Sexual epigenetics: genome-wide analysis revealed differential DNA methylation in the vector tick* Haemaphysalis longicornis*

**DOI:** 10.1186/s13071-025-06810-2

**Published:** 2025-06-01

**Authors:** Han Wang, Ziyan Bing, Lu Li, Ziwen Gao, Chuks Fidelis Nwanade, Na Dong, Ke Li, Leyan Du, Zhijun Yu

**Affiliations:** 1 Hebei Key Laboratory of Animal Physiology, Biochemistry and Molecular Biology, Hebei Collaborative Innovation Center for Eco-Environment, Ministry of Education Key Laboratory of Molecular and Cellular Biology, College of Life Sciences, Hebei Normal University, Shijiazhuang, 050024 China; 2 Guangdong Key Laboratory of Animal Conservation and Resource Utilization, Guangdong Public Laboratory of Wild Animal Conservation and Utilization, Institute of Zoology, Guangdong Academy of Sciences, Guangzhou, 510260 China

**Keywords:** *Haemaphysalis longicornis*, Sexual dimorphism, DNA methylation, Whole-genome bisulfite sequencing, Epigenetic regulation

## Abstract

**Background:**

*Haemaphysalis longicornis* is an important vector that transmits a variety of pathogens to humans and animals. This tick species is unique for having two separate reproductive populations: bisexual and parthenogenetic populations. In bisexual populations, morphological differences exist between the males and females, with the females often larger than the males. DNA methylation, as a key epigenetic modification, plays a crucial role in biological processes such as the maintenance of normal cellular function, the regulation of gene expression, and embryonic development. However, the epigenetic mechanism underlying sex differentiation in the bisexual population of *H. longicornis* has been overlooked.

**Methods:**

In the present study, the global DNA methylation profiles of the female and male *H. longicornis* ticks from the bisexual population were explored using whole-genome bisulfite sequencing. Differentially methylated regions (DMRs) were identified, followed by Gene Ontology (GO) and Kyoto Encyclopedia of Genes and Genomes (KEGG) pathway analysis of DMR-related genes.

**Results:**

The results revealed that DNA methylation levels in *H. longicornis* varied by sex and sequence context (CG, CHG, and CHH). The 3′ untranslated region (UTR) had the highest methylation in the CG context, followed by exons, introns, and CGI_shore regions. Female ticks generally exhibited higher methylation levels than males, particularly in gene body regions. A total of 10,460 DMRs were identified, with 5282 hypermethylated and 5178 hypomethylated. Further, GO and KEGG pathway analyses showed that differentially methylated genes (DMGs) were highly enriched in binding and metabolic pathways.

**Conclusions:**

These results broaden our understanding of DNA methylation changes associated with the female and male of *H. longicornis* and provide an important theoretical basis for subsequent studies of epigenetic mechanisms of sex differences in ticks.

**Graphical Abstract:**

Genome-wide DNA methylation analysis revealed epigenetic differences between male and female Haemaphysalis longicornis. Male and female ticks have significantly different methylation sites in multiple regions of the genome, and these sites may regulate gender specific biological functions. 
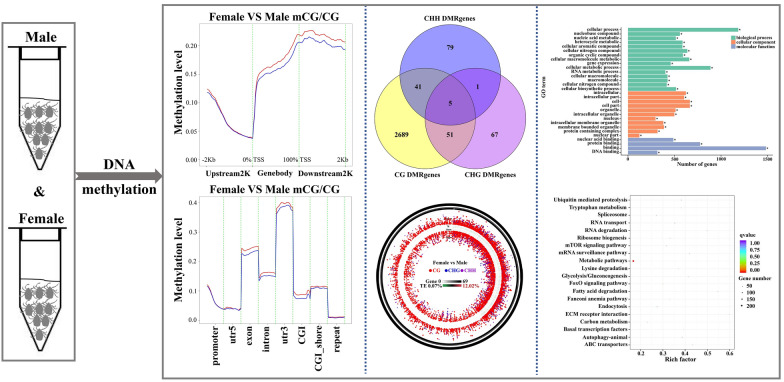

**Supplementary Information:**

The online version contains supplementary material available at 10.1186/s13071-025-06810-2.

## Background

Sexual dimorphism is a common phenomenon across a wide range of sexually reproducing organisms. It is often considered a diverse feature and refers to the behavioral, morphological (physical), and physiological differences between males and females [1, 2]. While some of these differences are driven by genes situated on the sex chromosomes [3], others have been associated with other genomic processes, such as epigenetic mechanisms and alternative splicing [4, 5]. These mechanisms contribute to the phenomenon known as sex-biased gene expression, which is widely recognized as a factor influencing most of the sex-specific differentiation [2].

DNA methylation is a crucial epigenetic modification involved in diverse biological processes [6]. Major forms include 5-methylcytosine (5mC) and *N*^6^-methyladenine (m^6^A) [7]. DNA methylation in insects occurs in the transcriptional regions of genes; the levels are generally lower than in vertebrates [8] and show great variation between arthropods [9]. DNA methyltransferases (DNMTs) catalyze the formation of 5mC by transferring methyl groups from S-adenosylmethionine (SAM) to the fifth carbon of cytosine residues [10]. DNA methylation regulates gene expression in both eukaryotes and prokaryotes and is involved in various biological processes including development, nutrigenomics, tumorigenesis, and DNA repair [10–12]. Despite some evidence suggesting the possible involvement of DNA methylation in sexual differentiation across various arthropods [13–15], differential DNA methylation patterns remain unclear in ticks. Nevertheless, recent research has found DNA methylation patterns in ticks in response to cold tolerance [7]. To advance our understanding in this area, additional research is needed to explore methylation patterns of male and female ticks. This is of significant interest since it can provide insights into the biological mechanisms behind tick sexual differences (sexual dimorphism). By manipulating these epigenetic processes, new approaches to controlling ticks and associated pathogens can potentially be developed.

Among globally distributed tick species, *Haemaphysalis longicornis* is native to East Asia but is considered an invasive species in regions such as Australia, New Zealand, and the United States [16, 17]. This tick consists of a diploid bisexual population, a triploid parthenogenetic population, and an aneuploid population capable of parthenogenetic and bisexual reproduction [18]. The parthenogenetic populations are found in Australia, New Zealand, Korea, Japan, the United States, and some areas in China (Sichuan and Shanghai), whereas the bisexual populations are widely distributed across China [19, 20]. The female *H. longicornis* can ingest a great amount of blood, whereas the males feed less and display remarkable morphological differences from females. This tick has been associated with a variety of human and animal pathogens and can transmit bacteria, fungi, viruses, and parasites [21]. These pathogens cause disease in humans, wildlife, and livestock, making them a potential threat to public and veterinary health [22]. Given their long life cycles and unique ecological adaptations, ticks may have evolved a highly complex set of epigenetic regulatory mechanisms. This can provide a strong adaptive response and buffer capacity in the changing natural environment, thus effectively ensuring the sustainability of its survival and reproduction process [23].

In this study, the DNA methylation status of adult males and females of *H. longicornis* was comprehensively analyzed using the whole-genome bisulfite sequencing (WGBS) technique, and sex specific DNA methylation profiles were assessed. These findings provide new insights into the epigenetic regulation of sex development in *H. longicornis*, and further expand our understanding of reproductive divergence and epigenetics in *H. longicornis*.

## Methods

### Ticks

Unfed adults of *H. longicornis* were collected from vegetation by flagging/dragging at the Xiaowutai Mountain National Nature Reserve Area, Hebei Province, China. For feeding, ticks were put into cloth bags attached to the ears of domestic rabbits [24]. Second-generation adults (20 females and 20 males) 2 weeks post-molting were randomly selected for the subsequent experiment, which was carried out three times. Cytological studies have found that the number of chromosomes of the bisexual tick was 21–22 [19], and from this, combined with stereoscopic and scanning electron microscopy observation of the morphology of female genital pores and Haller's organ [18], it was determined that the ticks used represented a bisexual population. During the unfed period, they were placed in an incubator (26 ± 1 °C, 75 ± 5% relative humidity [RH], 16:8 h [light/dark, L:D] photoperiod). All experiments involving rabbits were approved by the Animal Ethics Committee of Hebei Normal University (Protocol Number: IACUC-209230).

### DNA quantification and qualification

Genomic DNA was extracted using a Magnetic Universal Genomic DNA Kit (Tiangen Corporation, Beijing, China), following the manufacturer's recommendations. Genomic DNA degradation and contamination were validated by agarose gels. DNA purity was checked using a NanoPhotometer^®^ spectrophotometer (Implen, CA, USA). DNA concentration was measured using a Qubit^®^ DNA Assay Kit in a Qubit^®^ 2.0 Fluorometer (Life Technologies, CA, USA). DNA quality assessment data for the WGBS are listed in Table S1.

### Library preparation and quantification

A total of 100 ng genomic DNA spiked with 0.5 ng lambda DNA was fragmented by sonication to 200–300 base pairs (bp) with the Covaris S220 Focused-ultrasonicator. These DNA fragments were treated with bisulfite using the EZ DNA Methylation-Gold™ Kit (Zymo Research), and the library was constructed by Novogene Corporation (Beijing, China). Subsequently, paired-end sequencing of the sample was performed on the Illumina platform (Illumina, CA, USA). Library quality was assessed on an Agilent 2100 Bioanalyzer system.

### Data analysis

The library was sequenced on the Illumina NovaSeq platform. Image analysis and base calling were performed with the Illumina CASAVA pipeline, and finally, 150-bp paired-end reads were generated. The sequenced raw data have been deposited in the National Center for Biotechnology Information (NCBI) Sequence Read Archive (SRA) with accession number PRJNA938509.

### Quality control

First, basic statistics on the quality of the raw reads were performed using FastQC (fastqc_v0.11.5). Then, those read sequences produced by the Illumina pipeline in FASTQ format were pre-processed through fastp (fastp 0.20.0). The remaining reads that passed all the filtering steps were counted as clean reads, and all subsequent analyses were based on this. Finally, we used FastQC to perform basic statistics on the quality of the clean data reads.

### Reference data preparation before analysis

Prior to the analysis, the reference data for the species under study were prepared, which included the reference sequence in FASTA format, the annotation file in gtf format, the GO annotation file, the description file, and the gene region file in bed format. Repeats were predicted using RepeatMasker, and the CpG-island (CGI) track was generated from a genome using cpgIslandExt.

### Read mapping to the reference genome

Bismark software (version 0.16.3) was used to perform alignments of bisulfite-treated reads to a reference genome (–X 700 dovetail) [25]. The reference genome was first transformed into a bisulfite-converted version (C-to-T and G-to-A conversion) and then indexed using bowtie2 [26]. Clean reads were also transformed into fully bisulfite-converted versions (C-to-T and G-to-A conversion) before being aligned to the similarly converted versions of the genome in a directional manner. Sequence reads that produced a unique best alignment from the two alignment processes (original top and bottom strand) were then compared to the normal genomic sequence, and the methylation state of all cytosine positions was inferred. Reads that aligned to the same regions of the genome were regarded as duplicate reads. The sequencing depth and coverage were summarized using de-duplicated reads.

The results of the methylation extractor (bismark_methylation_extractor, – no_overlap) were transformed into bigWig format for visualization using the Integrative Genomics Viewer (IGV) browser. The sodium bisulfite non-conversion rate was calculated as the percentage of cytosine sequenced at cytosine reference positions in the lambda genome.

### Estimating methylation level

Methylated sites were identified with a binomial test using the methylated counts (mC), total counts (mC + unmethylated count [umC]), and the non-conversion rate (*r*). Sites with a false discovery rate (FDR)-corrected *P*-value < 0.05 were considered as a methylated site. To calculate the methylation level of the sequence, we divided the sequence into multiple bins, with a bin size of 10 kilobases (kb). The sum of methylated and unmethylated read counts in each window was calculated. The methylation level for each window or C site shows the fraction of mCs, and is defined as follows:

$$ {\text{ML}}\left( {\text{C}} \right) \, = {\text{reads}}\left( {{\text{mC}}} \right)/{\text{reads}}\left( {{\text{mC}}} \right) \, + {\text{ reads}}\left( {\text{C}} \right), $$

where ML is the methylation level.

### Differential methylation analysis

Differentially methylated regions (DMRs) were identified using Decision Support System (DSS) software [27–29]. The core of DSS is a new dispersion shrinkage method for estimating the dispersion parameter from gamma-Poisson or beta-binomial distributions. According to the distribution of DMRs through the genome, we defined the genes related to DMRs as genes whose gene body region (from transcription start site [TSS] to transcription end site [TES]) or promoter region (2 kb upstream from the TSS) overlapped with the DMRs.

### Gene Ontology (GO) and Kyoto Encyclopedia of Genes and Genomes (KEGG) enrichment analysis of DMR-related genes

GO enrichment analysis of genes related to DMRs was implemented using the GOseq R package [30], in which gene length bias was corrected. GO terms with corrected *P*-values less than 0.05 were considered significantly enriched by DMR-related genes. The KEGG Orthology-Based Annotation System (KOBAS) was utilized to assess the statistical enrichment of genes associated with DMRs within KEGG pathways [31].

## Results

### Whole-genome bisulfite sequencing and DNA methylation

In this study, to investigate the role of DNA methylation in the sexual dimorphism development of *H. longicornis*, WGBS technology was applied to sequence three repetitive sequences of female and male adult *H. longicornis*. During the sequencing process, female groups generated an average of 316,263,220 raw reads, while male groups generated 326,746,634. After quality control and filtering, approximately 309,318,166 reads in the female groups and 317,373,434 reads in the male groups were recognized as pure reads. Of these, 40.46% of the female sample reads and 39.44% of the male sample reads were uniquely mapped to the reference genome of the ticks, showing high sequencing quality and data accuracy. Repeat rates were similar between groups: 24.65% (females) and 24.57% (males). In addition, the bisulfite conversion rate exceeded 99.68% for both groups, which further demonstrated the high confidence of the WGBS data (Table 1).

**Table 1:** Summary of bisulfite (BS) sequencing of the tick *Haemaphysalis longicornis*

	Female	Male
Raw reads	316,263,220	326,746,634
Raw bases (G)	94.88	98.02
Clean reads	309,318,166	317,373,434
Clean bases (G)	84.65	86.39
Clean ratio (%)	89.22	88.14
Q20 (%)	97.10	96.67
Q30 (%)	91.09	90.19
GC content (%)	24.20	24.14
BS conversion rate (%)	99.68	99.68
Mapped reads	125,144,776	125,038,818
Mapping rate (%)	40.46	39.44
Duplicate rate (%)	24.65	24.57

In the analysis of DNA methylation levels, the proportion of methylated cytosines in the three sequence environments, CG, CHG, and CHH, was of particular interest. In female ticks, approximately 0.56% of the genomic C site was methylated, while in males, this proportion was slightly lower at 0.47%. In the CG sequence environment, the methylation level was 2.30% in females and 1.92% in males. It may be that more repetitive raw reads in the male sample were filtered, and fewer methylation sites were ultimately retained. In the CHG and CHH sequence environments, the methylation levels were almost the same between the two groups, 0.04% and 0.01%, respectively (Table 2).

**Table 2:** Proportion of methylated C sites occurring in different sequence environments

	Methylated C	Methylated C percentage	mC in CG	Methylated CG percentage	mC in CHG	Methylated CHG percentage	mC in CHH	Methylated CHH percentage
Female	8,414,041	0.56%	8,150,905	2.30%	127,071	0.04%	136,066	0.01%
Male	6,997,483	0.47%	6,744,365	1.92%	124,835	0.04%	128,287	0.01%

### Sample correlation and cluster analysis

**Fig. 1:**
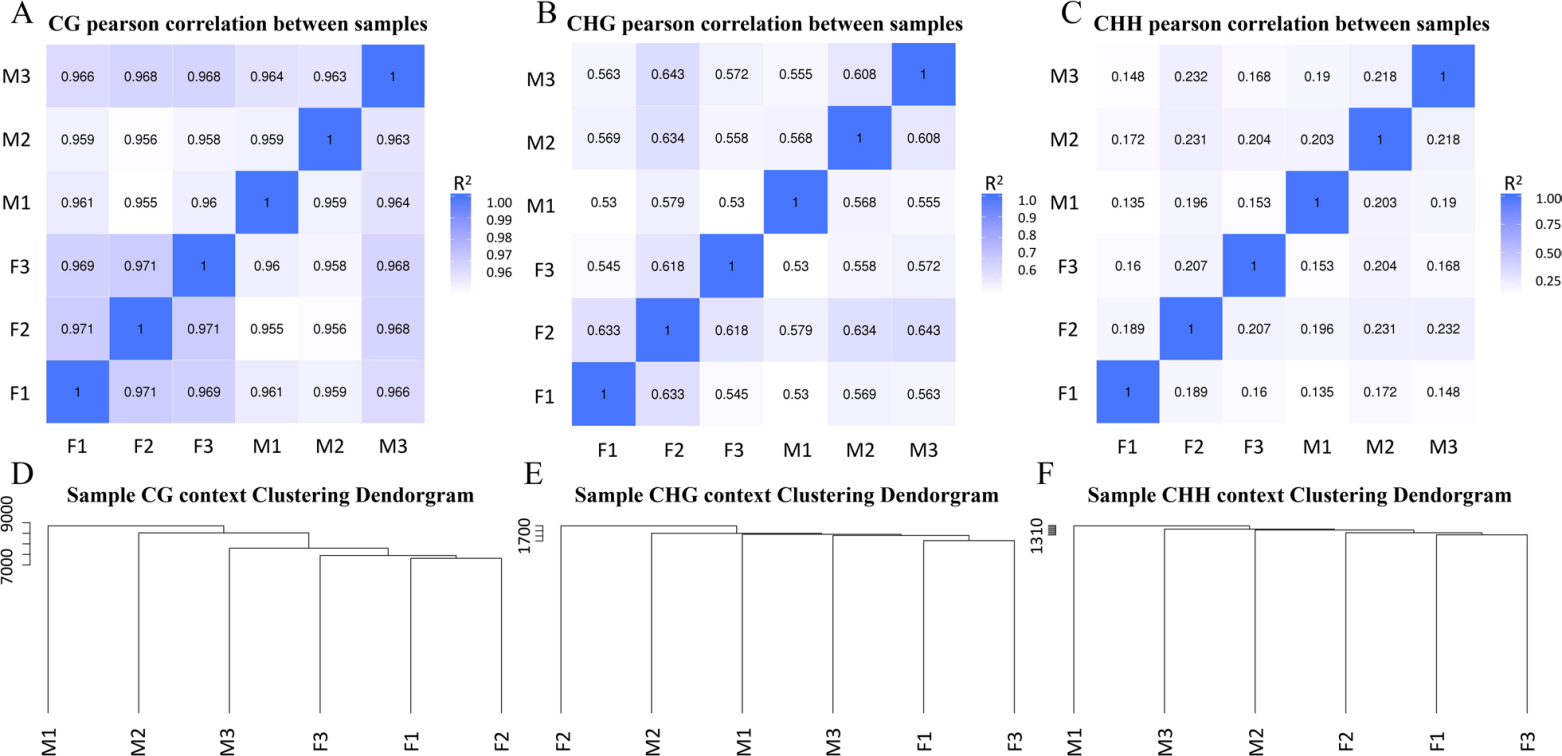
Pearson correlation based on the CG/CHG/CHH context among the replicate groups. **A** Pearson correlation based on CG context among the replicate groups. **B** Pearson correlation based on CHG context among the replicate groups. **C** Pearson correlation based on CHH context among the replicate groups. **D** Sample CG context clustering dendrogram. **E** Sample CHG context clustering dendrogram. **F** Sample CHH context clustering dendrogram. *R*^*2*^ Pearson's correlation coefficient; *F* female group; *M* male group

The correlation of methylation levels between groups was analyzed as a key indicator for assessing the reliability of the study. Pearson correlation analyses were performed on duplicate groups of female and male *H. longicornis* based on data in the CG, CHG, and CHH sequence environments. In the CG sequence environment, the Pearson correlation coefficients between both female and male groups exceeded 0.95, a result that indicates a strong correlation between the two groups of groups (Fig. 1A). In the CHG sequence environment, the Pearson correlation coefficients between the two groups of groups ranged from 0.5 to 0.7, which indicated a weak correlation between the two groups of groups (Fig. 1B). In contrast, the Pearson correlation coefficients of the two groups of groups in the CHH sequence environment ranged from 0.1 to 0.3, which indicated a lack of correlation between the two groups of groups (Fig. 1C).

When the groups were analyzed for clustering based on CG methylation patterns, males and females clustered separately (Fig. 1D). In the CHG sequence environment, a certain degree of clustering tendency could still be observed (Fig. 1E). Males and females also clustered separately in the CHH sequence environment (Fig. 1F).

### DNA methylation levels in functional regions

**Fig. 2:**
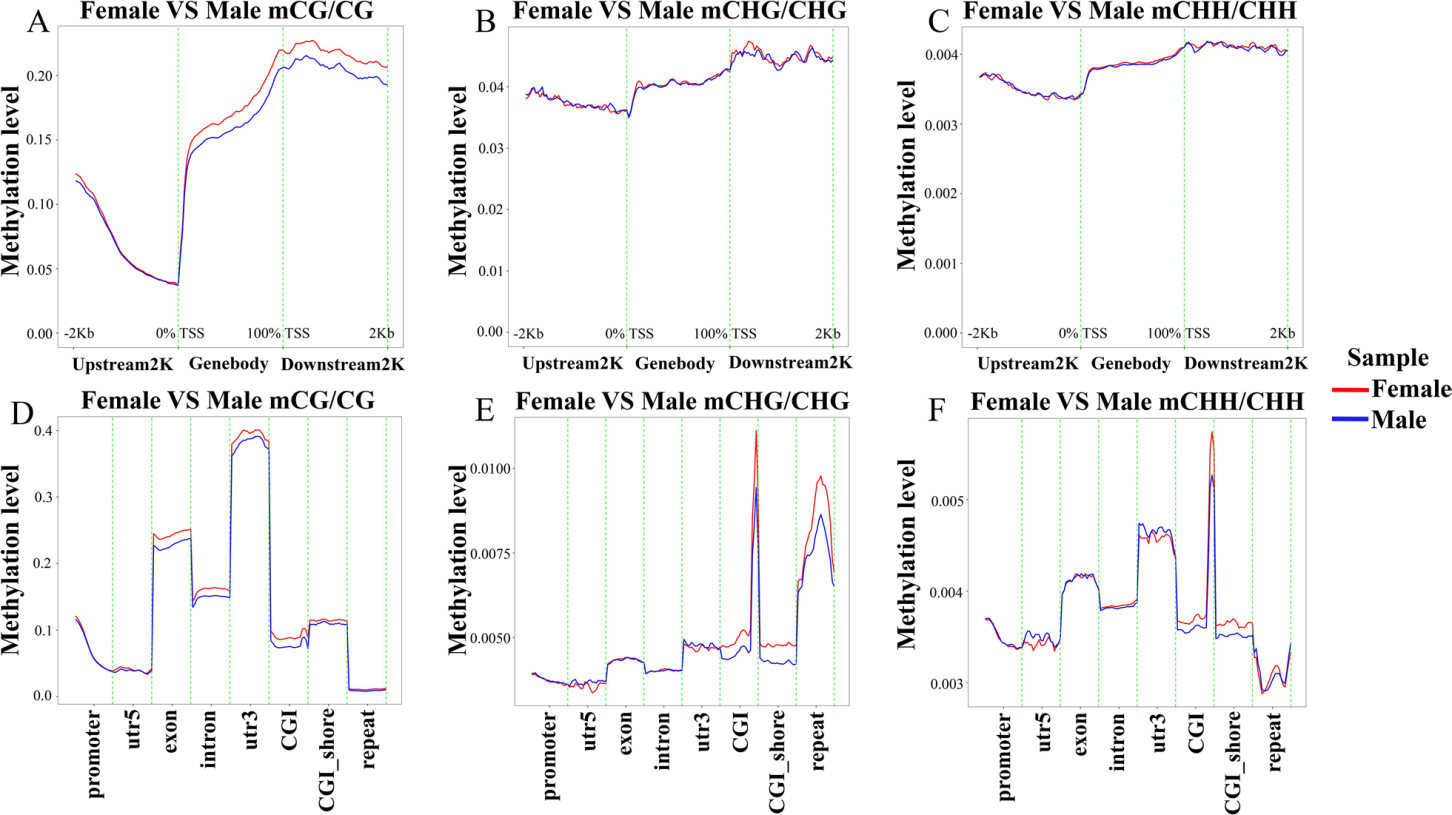
Distribution of methylation levels of functional regions, and the gene upstream and downstream between the female group and male group. **A** Distribution of methylation level of functional regions between female group and male group in mCG/CG. **B** Distribution of methylation level of functional regions between female group and male group in mCHG/CHG. **C** Distribution of methylation level of functional regions between female group and male group in mCHH/CHH. **D** Distribution of methylation level of genes upstream and downstream between female group and male group in mCG/CG. **E** Distribution of methylation level of genes upstream and downstream between female group and male group in mCHG/CHG. **F** Distribution of methylation level of genes upstream and downstream between female group and male group in mCHH/CHH. *TSS* transcription start site, *TES* transcription end site

**Fig. 3:**
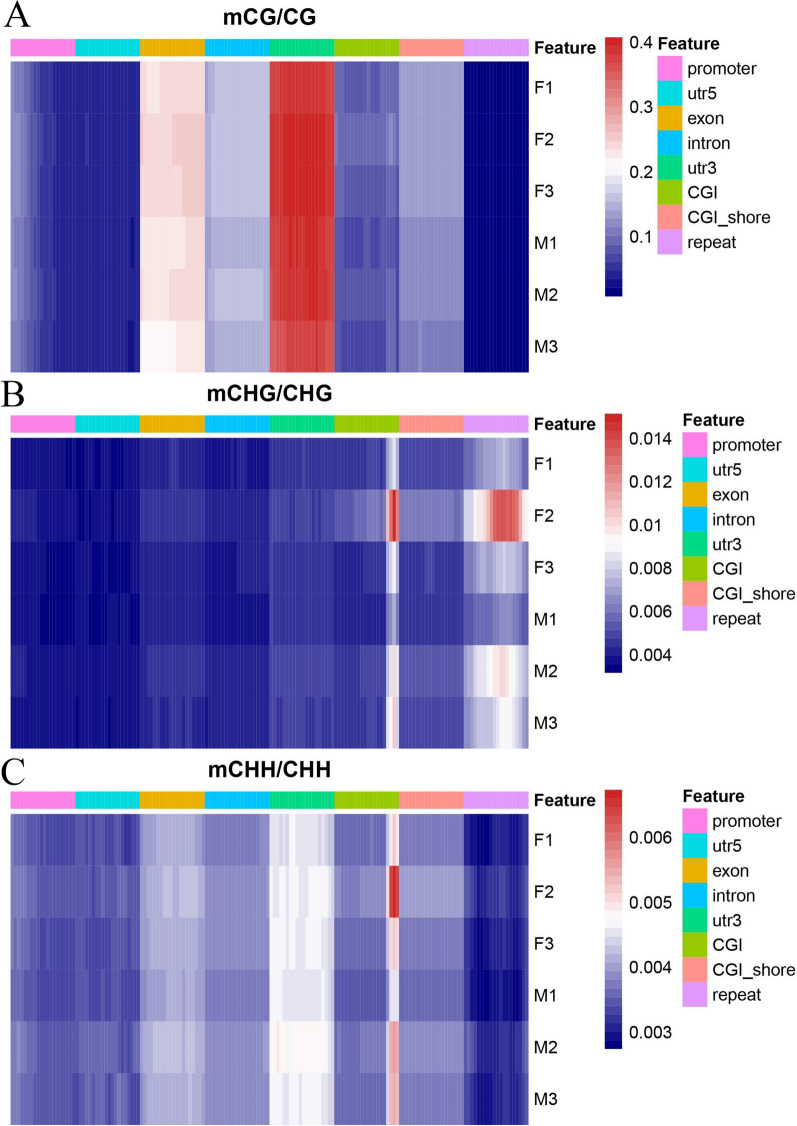
Heat map of differences in methylation levels in different gene regions. **A** Heat map of differences in methylation levels in mCG/CG. **B** Heat map of differences in methylation levels in mCHG/CHG. **C** Heat map of differences in methylation levels in mCHH/CHH

Differences in methylation levels were assessed in three sequence environments, CG, CHG, and CHH, as well as in different gene regions (DMRs). The results showed that within the 2-kb region upstream of the TSS, the differences in methylation levels between the two groups were not significant. However, in the gene body region and downstream 2-kb region, the methylation level of female groups was slightly higher than that of male groups, and the peak methylation appeared in the downstream region near the TES (Fig. 2A). Figure 2B and C demonstrate that there was little difference in methylation levels between the sexes in the CHG and CHH sequence environments. In the differential analysis of methylation levels in different gene regions, the 3′ untranslated region (UTR) had the highest methylation level in the CG sequence environment, followed by exon, intron, and CGI_shore, and the methylation levels of female groups were generally higher than those of male groups. In contrast, the methylation levels of the 5′ UTR and repeat regions were relatively low (Fig. 2D). In the CHG and CHH sequence environments, the highest methylation levels were found in the CGI region and were higher in female groups than in male groups. In the repeat region, the CHG type had a higher methylation level, while the CHH type had the lowest methylation level, a finding that is reflected in Fig. 2E and F. To demonstrate these differences more visually, heat maps were used to show the differences in methylation levels in CG, CHG, and CHH sequence environments in DMRs (Fig. 3).

### Analysis of DMRs

To further elucidate the role of DNA methylation in the sexual dimorphism development of *H. longicornis*, an analysis was conducted to identify DMRs and differentially methylated genes (DMGs) in adult females and males. A total of 10,460 DMRs were identified in female and male groups (Table S2). These DMRs were further classified into 5282 hypermethylated DMRs and 5178 hypomethylated DMRs, and the specific information is listed in Tables S3 and S4, respectively.

**Fig. 4:**
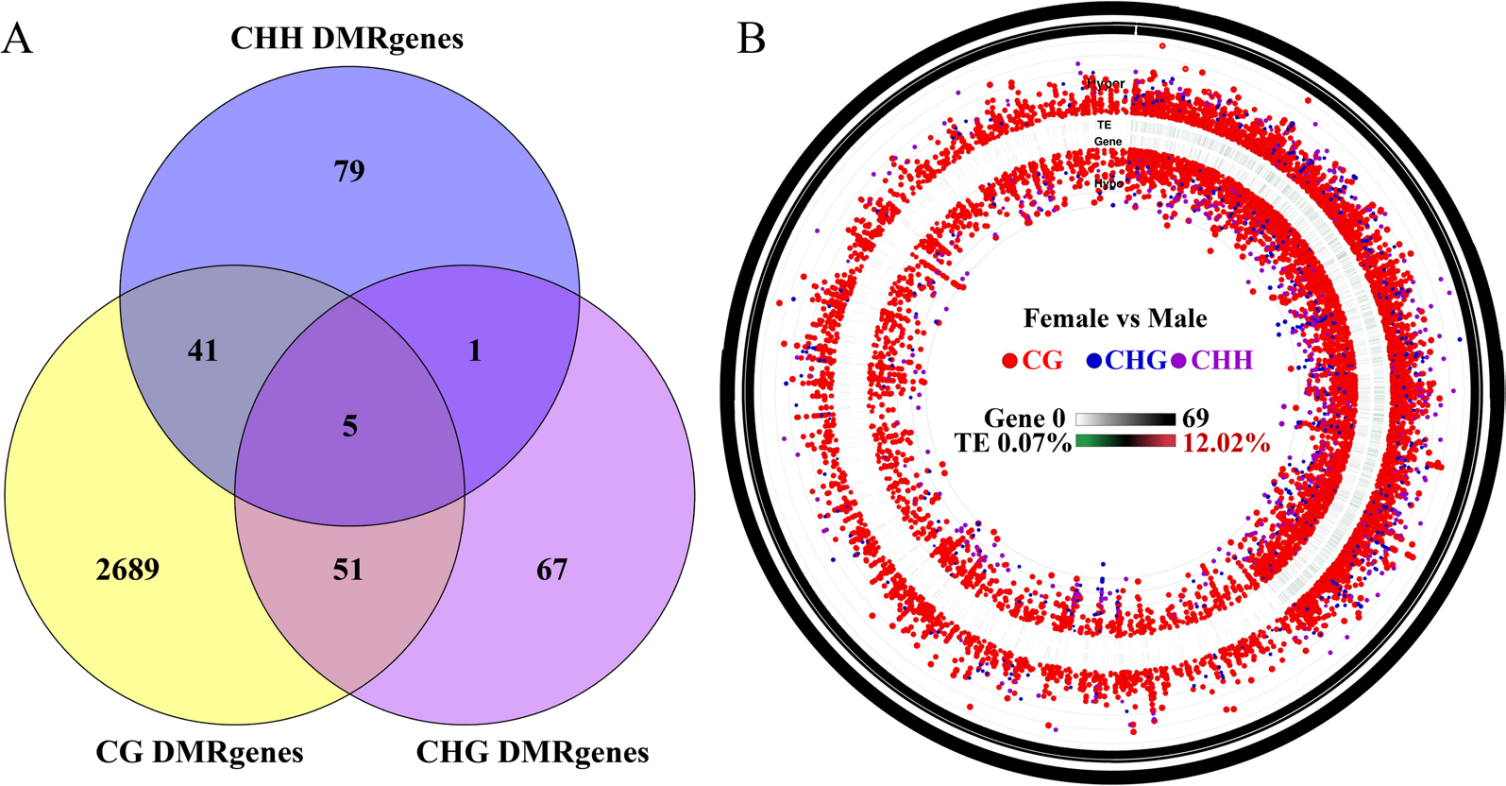
The methylation patterns between the female group and male group of *H. longicornis.* **A** Venn diagrams of female and male group DMGs under CG, CHG, and CHH contexts of the *H. longicornis* adults. **B** Significantly different methylation patterns between the female group and male group

**Fig. 5:**
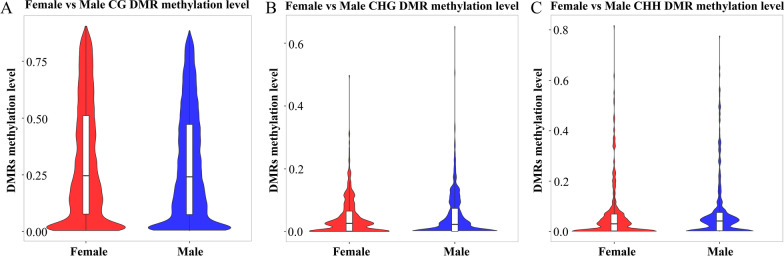
Violin plot analysis of the methylation level of DMRs in the female and male groups of *H. longicornis* adults. **A** Violin plot analysis of CG DMR methylation level. **B** Violin plot analysis of CHG DMR methylation level. **C** Violin plot analysis of CHH DMR methylation level

The DMGs in the context of CG, CHG, and CHH are demonstrated by Venn diagrams. The analysis revealed that 2786, 124, and 126 DMGs were observed in CG, CHG, and CHH sequence environments, respectively, with five DMGs exhibiting differential methylation in all three sequence environments (Fig. 4A). Furthermore, the number of red dots indicates that the most methylation occurs at the CG locus between females and males, while noting that the expression levels of transposons ranged from 0.07% to 12.02% (Fig. 4B). In addition, a violin plot analysis of methylation levels in the DMRs was performed. The results showed that the methylation level was highest in the CG sequence environment. The median value of the DMR methylation level associated with the female groups was slightly higher than that of male groups in the CG and CHH sequence environments, but lower than that of the male group in the CHG (Fig. 5).

### GO and KEGG pathway enrichment analysis

**Fig. 6:**
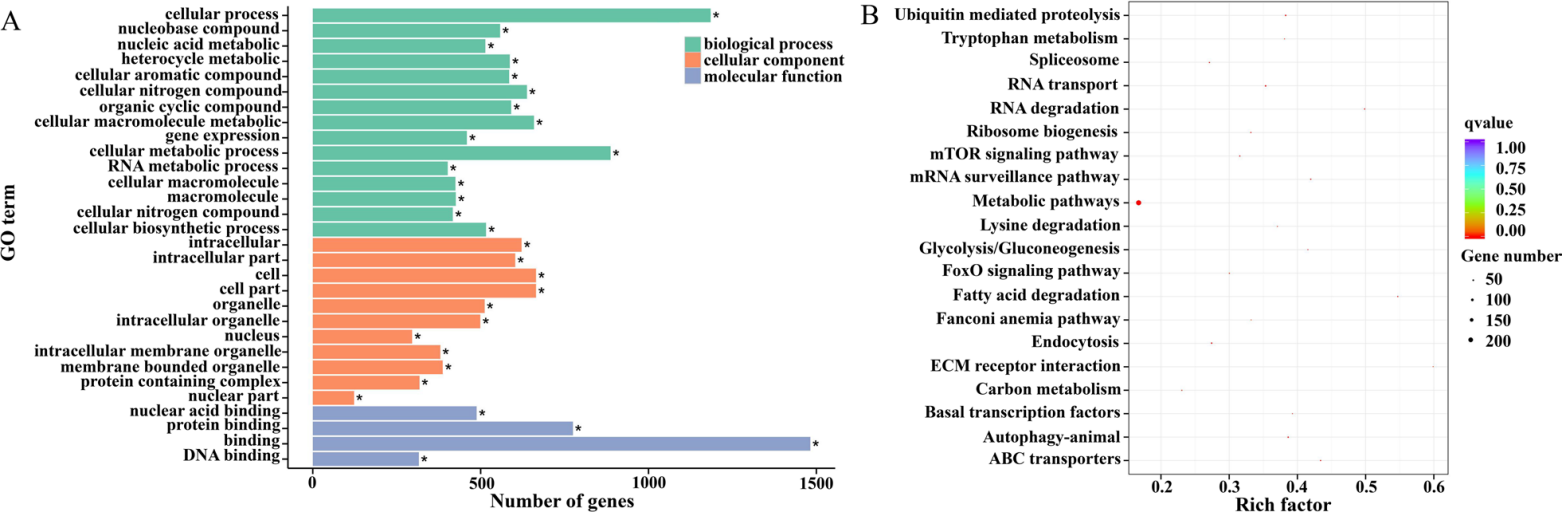
GO and KEGG enrichment analyses of the female and male group CG DMGs of the *H. longicornis* adults. **A** GO enrichment analysis of DMGs. **B** KEGG pathway enrichment

GO and KEGG analyses were performed to investigate DNA methylation changes in *H. longicornis* across sexes. Given that the majority of DMGs are located in CG methylation backgrounds, DMG functional enrichment analyses were performed based on CG methylation. GO enrichment analysis was classified into three main categories: biological processes, cellular components, and molecular functions. The results of the analyses showed that biological processes were mainly enriched in cellular processes. The cellular components were mainly enriched in the cell, cell part, and intracellular region, while the molecular functions were primarily enriched in binding (Fig. 6A). In addition, KEGG pathway enrichment analysis was performed on DMGs. The results showed that KEGG pathways were mainly enriched in metabolic pathways (Fig. 6B). These findings help to reveal the biological significance and potential molecular mechanisms of DNA methylation changes in *H. longicornis* in different sexes.

## Discussion

Modifications to DNA and histones affect changes in chromatin and the expression of genes that regulate many cellular and developmental processes. DNA methylation plays an important role in regulating a variety of biological processes, such as social insect behavior and population differentiation [32]. Preliminary studies on honeybees suggest that DNA methylation is instrumental in regulating population differentiation and memory processing behavior [33, 34]. Knockdown of the DNA methyltransferase 3 (Dnmt3) gene in honeybees resulted in reduced genome-wide methylation levels and altered patterns of gene RNA splicing, suggesting that Dnmt3 and DNA methylation play an important role in queen differentiation [35]. Studies have shown that king and worker larvae have significantly different methylation patterns, demonstrating that DNA methylation is important in termite *Zootermopsis nevadensis* grade differentiation [36]. DMGs between populations have been identified in the reproductive and sterile bumblebee workers [37]. In the ants *Camponotus floridanus* and *Harpegnathos saltator*, there is a link between DNA methylation and RNA splicing [38]. Dnmt methyltransferases involved in the de novo methylation of tick DNA have been identified in previous studies [39, 40]. However, the role of DNA methylation in the sexual dimorphism development and physiological regulation of the tick has not been clearly defined. The two breeding populations of *H. longicornis*, the bisexual population and the parthenogenetic population, have different DNA methylation transcription patterns at different life stages [41]. In this study, WGBS was used for the first time to explore DNA methylation changes in a bisexual population of *H. longicornis*. This allowed us to comprehensively study genome-wide DNA methylation profiles in the bisexual population.

The results of the present study showed that the methylated C sites of *H. longicornis* female ticks were slightly higher than in male ticks. According to existing research, there are differences in DNA methylation based on sex. While some studies have reported higher methylation levels in females [42], others have found higher methylation levels in males [5, 43] or similar methylation between males and females [2]. These contradictory results across various studies may be attributed to many factors, including the techniques used for DNA methylation analysis, sample size, and the parts or tissues analyzed, indicating the complexity of sex-specific DNA methylation. The slightly higher methylation of the female tick may correlate with females typically becoming larger (engorged) than males after a blood meal, which is essential for egg production and development. In addition, we observed that the methylated genomic C site in the males (0.47%) and females (0.56%) of *H. longicornis* was lower when compared with a previous study of adult females, which found levels of 0.95% and 0.94% in the control and low-temperature-treated groups, respectively [7]. This demonstrates the importance of exploring DNA methylation in individual species, since methylation can vary within species and/or between sexes, and many factors, such as cell or tissue types, age, and environmental conditions, can result in varying degrees of methylation. Our results also implied that methylation (methylated genomic C site) in ticks, including *H. longicornis*, is low (less than 1%), although much higher than that in some insects [7].

Based on the sequence of cytosines (CG, CHG, and CHH), we found that the methylation levels in the CG sequence environment were higher in females than males, and levels were almost the same between the two groups in the CHG and CHH sequence environments. This suggests that the different methylation levels across these sequences may play a role in sex-specific differences and that high CG methylation may be associated with sex differences, contributing to the biological characteristics between *H. longicornis* males and females at the molecular level. Males and females of other arthropods are known to have differential and/or variable CpG methylation patterns [43, 44], which is a potential mechanism underlying sex-specific differences. Our results also suggest that methylation in ticks, including *H. longicornis*, occurs at the CG site, which is consistent with a previous DNA methylation study of *H. longicornis* exposed to low-temperature stress [7]. Additionally, our cluster analysis revealed that male and female groups of the studied ticks clustered separately, which was more pronounced in CG and CHH sequence environments, possibly indicating where each group exhibits a specific methylation profile. These results highlight a promising direction for future studies.

By analyzing the DNA methylation levels in different genomic functional regions, we found that the differences showed sex-specific and region/site dependence. For instance, the 3′ UTR region had the highest methylation level in the CG sequence environment, followed by the exon, intron, and CGI_shore regions, suggesting that these regions may have important roles in the regulation of gene expression. It has also been shown that *H. longicornis* has different functional genomic regions at the CG site, and DNA methylation is more likely to occur in the 3’ UTR region [7]. Moreover, the female groups generally had higher methylation levels than the male groups, especially in the gene body region and downstream regions near the TES, which may affect gene expression and function. Indeed, DNA methylation within the gene body region usually results in higher levels of gene expression, but methylation in other regions/sites may have the opposite effect [45–47]. This situation is often referred to as the “DNA methylation paradox,” where methylation in different sites/regions has opposite effects on a gene, and the specific location of methylation can determine whether the gene is silenced or actively transcribed [47]. Overall, the DNA methylation patterns differed slightly between female and male ticks. Our results suggest that CG methylation may play an important role in the gene regulation of *H. longicornis*, particularly in a sex-specific context, acting as a key epigenetic mechanism that determines which genes may be expressed and/or repressed in the males and females of this tick species. However, how the different genomic regions or sites (sequence environments) contribute to gene regulation remains unclear, making further exploration necessary.

To further elucidate the role of DNA methylation in the sexual differentiation of *H. longicornis*, analysis was conducted to identify DMRs and DMGs in adult female and male groups. Overall, we found differentially hypermethylated and hypomethylated regions between the two groups, and a greater number of genes were differentially methylated at the CG, which further demonstrates differential methylation based on sex and regions/sites (sequence environments). DMGs in *H. longicornis* are involved in several biological processes, cellular components, and molecular functions, and DMGs are significantly enriched in binding and RNA transport during cold stress [7]. Here, our GO and KEGG pathway analyses revealed that DMGs were most significantly enriched in binding and metabolic pathways, suggesting their potential involvement in regulating relevant genes in the males and females of *H. longicornis*. However, additional studies are needed to gain a comprehensive understanding of their contributions to the regulation of relevant genes and to examine the expression pattern of genes related to males and females of *H. longicornis*, which will provide exciting new directions for further investigation.

## Conclusions

DNA methylation profiles of adult male and female *H. longicornis* were comprehensively analyzed. Different methylation patterns were found between the sexes, which may affect gene expression related to sexual development. Sex-linked DMRs and DMGs were also identified. Before any conclusion can be drawn regarding the findings of this study, it is imperative to refine the mechanisms and functions of DNA methylation and its broader implications in the sexual differentiation of ticks, including *H. longicornis*. Nevertheless, these results may provide new clues and targets for the analysis of the mechanism of DNA methylation in the regulation of sex differences and the identification of key genes in ticks.

## Supplementary Information

Additional file 1.

Additional file 2.

Additional file 3.

Additional file 4.

## Data Availability

The sequenced raw data have been deposited in the National Center for Biotechnology Information (NCBI) Sequence Read Archive (SRA) with the accession number PRJNA938509.

## References

[1] Wan ZY, Xia JH, Lin G, Wang L, Lin VC, Yue GH. Genome-wide methylation analysis identified sexually dimorphic methylated regions in hybrid tilapia. Sci Rep. 2016;6:35903. 10.1038/srep35903. 27782217 10.1038/srep35903 PMC5080608

[2] Yu X, Marshall H, Liu Y, Xiong Y, Zeng X, Yu H, et al. Sex-specific transcription and DNA methylation landscapes of the Asian citrus psyllid, a vector of huanglongbing pathogens. Evolution. 2023;77:1203–15. 10.1093/evolut/qpad036. 36869727 10.1093/evolut/qpad036

[3] Mank JE. Sex chromosomes and the evolution of sexual dimorphism: lessons from the genome. Am Nat. 2009;173:141–50. 10.1086/595754. 20374139 10.1086/595754

[4] Wexler J, Delaney EK, Belles X, Schal C, Wada-Katsumata A, Amicucci MJ, et al. Hemimetabolous insects elucidate the origin of sexual development via alternative splicing. eLife. 2019;8:e47490. 10.7554/eLife.47490. 31478483 10.7554/eLife.47490 PMC6721801

[5] Bain SA, Marshall H, de la Filia AG, Laetsch DR, Husnik F, Ross L. Sex-specific expression and DNA methylation in a species with extreme sexual dimorphism and paternal genome elimination. Mol Ecol. 2021;30:5687–703. 10.1111/mec.15842. 33629415 10.1111/mec.15842

[6] Li H, Zhang N, Wang Y, Xia S, Zhu Y, Xing C, et al. DNA N6-methyladenine modification in eukaryotic genome. Front Genet. 2022;13:914404. 10.3389/fgene.2022.914404. 35812743 10.3389/fgene.2022.914404 PMC9263368

[7] Nwanade CF, Wang Z, Bai R, Wang R, Zhang T, Liu J, et al. DNA methylation variation is a possible mechanism in the response of *Haemaphysalis longicornis* to low-temperature stress. Int J Mol Sci. 2022;23:15207. 10.3390/ijms232315207. 36499526 10.3390/ijms232315207 PMC9740864

[8] Bewick AJ, Vogel KJ, Moore AJ, Schmitz RJ. Evolution of DNA methylation across insects. Mol Biol Evol. 2017;34:654–65. 10.1093/molbev/msw264. 28025279 10.1093/molbev/msw264 PMC5400375

[9] Yu Z, Pei T, Shi X, Nwanade CF, Bing Z, Gao Z, et al. The functions of DNA methyltransferases during the feeding and development of *Haemaphysalis longicornis* are potentially associated with lysosome pathways. BMC Gen. 2024;25:1109. 10.1186/s12864-024-11049-9. 10.1186/s12864-024-11049-9 PMC11577950 39563221

[10] Kausar S, Abbas MN, Cui H. A review on the DNA methyltransferase family of insects: aspect and prospects. Int J Biol Macromol. 2021;186:289–302. 10.1016/j.ijbiomac.2021.06.205. 34237376 10.1016/j.ijbiomac.2021.06.205

[11] Kucharski R, Maleszka J, Foret S, Maleszka R. Nutritional control of reproductive status in honeybees via DNA methylation. Science. 2008;319:1827–30. 10.1126/science.1153069. 18339900 10.1126/science.1153069

[12] Bergman Y, Cedar H. DNA methylation dynamics in health and disease. Nat Struct Mol Biol. 2013;20:274–81. 10.1038/nsmb.2518. 23463312 10.1038/nsmb.2518

[13] Zhang M, Chen J, Zhou X, Liang S, Li G, Wang F. Different genomic DNA methylation patterns between male and female adults of white-backed planthoppers *Sogatella furcifera*. J Asia-Pac Entomol. 2014;17:917–21. 10.1016/j.aspen.2014.10.007.

[14] Mathers TC, Mugford ST, Percival-Alwyn L, Chen Y, Kaithakottil G, Swarbreck D, et al. Sex-specific changes in the aphid DNA methylation landscape. Mol Ecol. 2019;28:4228–41. 10.1111/mec.15216. 31472081 10.1111/mec.15216 PMC6857007

[15] Hunt BJ, Pegoraro M, Marshall H, Mallon EB. A role for DNA methylation in bumblebee morphogenesis hints at female-specific developmental erasure. Insect Mol Biol. 2024;33:481–92. 10.1111/imb.12897. 38348493 10.1111/imb.12897

[16] Zhao L, Li J, Cui X, Jia N, Wei J, Xia L, et al. Distribution of *Haemaphysalis longicornis* and associated pathogens: analysis of pooled data from a China field survey and global published data. Lancet Planety Health. 2020;4:e320–9. 10.1016/S2542-5196(20)30145-5. 10.1016/S2542-5196(20)30145-5 32800150

[17] Yu Z, He B, Gong Z, Liu Y, Wang Q, Yan X, et al. The new *Haemaphysalis longicornis* genome provides insights into its requisite biological traits. Genomics. 2022;114:110317. 10.1016/j.ygeno.2022.110317. 35189284 10.1016/j.ygeno.2022.110317

[18] Chen Z, Yang X, Bu F, Yang X, Liu J. Morphological, biological and molecular characteristics of bisexual and parthenogenetic *Haemaphysalis longicornis*. Vet Parasitol. 2012;189:344–52. 10.1016/j.vetpar.2012.04.021. 22560314 10.1016/j.vetpar.2012.04.021

[19] Chen X, Xu S, Yu Z, Guo L, Yang S, Liu L, et al. Multiple lines of evidence on the genetic relatedness of the parthenogenetic and bisexual *Haemaphysalis longicornis* (Acari: Ixodidae). Infect Genet Evol. 2014;21:308–14. 10.1016/j.meegid.2013.12.002. 24316292 10.1016/j.meegid.2013.12.002

[20] Schappach BL, Krell RK, Hornbostel VL, Connally NP. Exotic *Haemaphysalis longicornis* (Acari: Ixodidae) in the United States: biology, ecology, and strategies for management. J Integ Pest Manag. 2020;11:21. 10.1093/jipm/pmaa019.

[21] Feng T, Tong H, Zhang Q, Ming Z, Song Z, Zhou X, et al. Targeting *Haemaphysalis longicornis* serpin to prevent tick feeding and pathogen transmission. Insect Sci. 2024;31:694–706. 10.1111/1744-7917.13260. 37635449 10.1111/1744-7917.13260

[22] Ye RZ, Li YY, Xu DL, Wang BH, Wang XY, Zhang MZ, et al. Virome diversity shaped by genetic evolution and ecological landscape of *Haemaphysalis longicornis*. Microbiome. 2024;12:35. 10.1186/s40168-024-01753-9. 38378577 10.1186/s40168-024-01753-9 PMC10880243

[23] De S, Kitsou C, Sonenshine DE, Pedra JHF, Fikrig E, Kassis JA, et al. Epigenetic regulation of tick biology and vectorial capacity. Trends Genet. 2021;37:8–11. 10.1016/j.tig.2020.09.012. 33020021 10.1016/j.tig.2020.09.012 PMC8008791

[24] Wang H, Bai R, Pei T, Meng J, Nwanade CF, Zhang Y, et al. Aquaporins modulate the cold response of *Haemaphysalis longicornis* via changes in gene and protein expression of fatty acids. Parasit Vectors. 2025;18:70. 10.1186/s13071-025-06718-x. 39994701 10.1186/s13071-025-06718-x PMC11849292

[25] Krueger F, Andrews SR. Bismark: a flexible aligner and methylation caller for Bisulfite-Seq applications. Bioinformatics. 2011;27:1571–2. 10.1093/bioinformatics/btr167. 21493656 10.1093/bioinformatics/btr167 PMC3102221

[26] Langmead B, Salzberg SL. Fast gapped-read alignment with Bowtie 2. Nat Methods. 2012;9:357–9. 10.1038/nmeth.1923. 22388286 10.1038/nmeth.1923 PMC3322381

[27] Feng H, Conneely KN, Wu H. A Bayesian hierarchical model to detect differentially methylated loci from single nucleotide resolution sequencing data. Nucleic Acids Res. 2014;42:e69. 10.1093/nar/gku154. 24561809 10.1093/nar/gku154 PMC4005660

[28] Wu H, Xu T, Feng H, Chen L, Li B, Yao B, et al. Detection of differentially methylated regions from whole-genome bisulfite sequencing data without replicates. Nucleic Acids Res. 2015;43:e141. 10.1093/nar/gkv715. 26184873 10.1093/nar/gkv715 PMC4666378

[29] Park Y, Wu H. Differential methylation analysis for BS-seq data under general experimental design. Bioinformatics. 2016;32:1446–53. 10.1093/bioinformatics/btw026. 26819470 10.1093/bioinformatics/btw026 PMC12157722

[30] Young MD, Wakefield MJ, Smyth GK, Oshlack A. Gene ontology analysis for RNA-seq: accounting for selection bias. Genome Biol. 2010;11:R14. 10.1186/gb-2010-11-2-r14. 20132535 10.1186/gb-2010-11-2-r14 PMC2872874

[31] Mao X, Cai T, Olyarchuk JG, Wei L. Automated genome annotation and pathway identification using the KEGG Orthology (KO) as a controlled vocabulary. Bioinformatics. 2005;21:3787–93. 10.1093/bioinformatics/bti430. 15817693 10.1093/bioinformatics/bti430

[32] Palli SR. Epigenetic regulation of post-embryonic development. Curr Opin Insect Sci. 2021;43:63–9. 10.1016/j.cois.2020.09.011. 33068783 10.1016/j.cois.2020.09.011 PMC8044252

[33] Lyko F, Foret S, Kucharski R, Wolf S, Falckenhayn C, Maleszka R. The honey bee epigenomes: differential methylation of brain DNA in queens and workers. PLoS Biol. 2010;8:e1000506. 10.1371/journal.pbio.1000506. 21072239 10.1371/journal.pbio.1000506 PMC2970541

[34] Lockett GA, Helliwell P, Maleszka R. Involvement of DNA methylation in memory processing in the honey bee. NeuroReport. 2010;21:812–6. 10.1097/WNR.0b013e32833ce5be. 20571459 10.1097/WNR.0b013e32833ce5be

[35] Foret S, Kucharski R, Pellegrini M, Feng S, Jacobsen SE, Robinson GE, et al. DNA methylation dynamics, metabolic fluxes, gene splicing, and alternative phenotypes in honey bees. P Nat Acad Sci USA. 2012;109:4968–73. 10.1073/pnas.1202392109. 10.1073/pnas.1202392109 PMC3324026 22416128

[36] Glastad KM, Gokhale K, Liebig J, Goodisman MA. The caste- and sex-specific DNA methylome of the termite *Zootermopsis nevadensis*. Sci Rep. 2016;6:37110. 10.1038/srep37110. 27848993 10.1038/srep37110 PMC5111047

[37] Marshall H, Lonsdale ZN, Mallon EB. Methylation and gene expression differences between reproductive and sterile bumblebee workers. Evol Lett. 2019;3:485–99. 10.1002/evl3.129. 31636941 10.1002/evl3.129 PMC6791180

[38] Bonasio R, Li Q, Lian J, Mutti NS, Jin L, Zhao H, et al. Genome-wide and caste-specific DNA methylomes of the ants *Camponotus floridanus* and *Harpegnathos saltator*. Curr Biol. 2012;22:1755–64. 10.1016/j.cub.2012.07.042. 22885060 10.1016/j.cub.2012.07.042 PMC3498763

[39] Yu Z, Pei T, Shi X, Nwanade CF, Bing Z, Gao Z, et al. The functions of DNA methyltransferases during the feeding and development of *Haemaphysalis longicornis* are potentially associated with lysosome pathways. BMC Genom. 2024;25:1109. 10.1186/s12864-024-11049-9. 10.1186/s12864-024-11049-9 PMC11577950 39563221

[40] Agwunobi DO, Zhang M, Shi X, Zhang S, Zhang M, Wang T, et al. DNA methyltransferases contribute to cold tolerance in ticks *Dermacentor silvarum* and *Haemaphysalis longicornis* (Acari: Ixodidae). Front Vet Sci. 2021;8:726731. 10.3389/fvets.2021.726731. 34513977 10.3389/fvets.2021.726731 PMC8426640

[41] Wang T, Wang T, Zhang M, Shi X, Zhang M, Wang H, et al. The ovarian development genes of bisexual and parthenogenetic *Haemaphysalis longicornis* evaluated by transcriptomics and proteomics. Front Vet Sci. 2021;8:783404. 10.3389/fvets.2021.783404. 34977217 10.3389/fvets.2021.783404 PMC8714755

[42] Grant OA, Wang Y, Kumari M, Zabet NR, Schalkwyk L. Characterising sex differences of autosomal DNA methylation in whole blood using the Illumina EPIC array. Clin Epigenet. 2022;14:62. 10.1186/s13148-022-01279-7. 10.1186/s13148-022-01279-7 PMC9107695 35568878

[43] Brink K, Thomas CL, Jones A, Chan TW, Mallon EB. Exploring the ageing methylome in the model insect, *Nasonia vitripennis*. BMC Gen. 2024;25:305. 10.1186/s12864-024-10211-7. 10.1186/s12864-024-10211-7 PMC10958858 38519892

[44] Field LM, Lyko F, Mandrioli M, Prantera G. DNA methylation in insects. Insect Mol Biol. 2004;13:109–15. 10.1111/j.0962-1075.2004.00470.x. 15056357 10.1111/j.0962-1075.2004.00470.x

[45] Moore LD, Le T, Fan G. DNA methylation and its basic function. Neuropsychopharmacology. 2012;38:23–38. 10.1038/npp.2012.112. 22781841 10.1038/npp.2012.112 PMC3521964

[46] Jang HS, Shin WJ, Lee JE, Do JT. CpG and non-CpG methylation in epigenetic gene regulation and brain function. Genes. 2017;8:148. 10.3390/genes8060148. 28545252 10.3390/genes8060148 PMC5485512

[47] Wang Q, Xiong F, Wu G, Liu W, Chen J, Wang B, et al. Gene body methylation in cancer: molecular mechanisms and clinical applications. Clin Epigenet. 2022;14:154. 10.1186/s13148-022-01382-9. 10.1186/s13148-022-01382-9 PMC9706891 36443876

